# Genome of *Helicobacter pylori* and Serotype of HPV Detected in Oropharyngeal and Laryngeal Cancer and Chronic Inflammation Patients

**DOI:** 10.3390/ijerph18189545

**Published:** 2021-09-10

**Authors:** Jaromír Astl, Richard Holy, Eva Maute, Jan Rotnágl, David Kalfeřt, Barbora Drnková, Temoore Younus, Emil Pavlík

**Affiliations:** 1 Department of Otorhinolaryngology and Maxilofacial Surgery, Military University Hospital Prague, 16902 Prague, Czech Republic; jaromir.astl@uvn.cz (J.A.); jan.rotnagl@uvn.cz (J.R.); 2 Third Faculty of Medicine, Charles University, 10000 Prague, Czech Republic; temoore.younus@lf3.cuni.cz; 3 Department of Otolaryngology Education, Institute of Postgradual Medical Education, 10005 Prague, Czech Republic; 4 Maute HNO-Praxis, 85276 Pfaffenhofen an der Ilm, Germany; praxis@hno-maute.de; 5 Department of Otorhinolaryngology and Head and Neck Surgery, University Hospital Motol, 15006 Prague, Czech Republic; david.kalfert@fnmotol.cz; 6 First Faculty of Medicine, Charles University, 12108 Prague, Czech Republic; barbora.drnkova@fbmi.cvut.cz (B.D.); emil.pavlik@vfn.cz (E.P.); 7 Faculty of Biomedical Engineering, Czech Technical University in Prague, 27201 Kladno, Czech Republic; 8 Department of Immunology and Microbiology and Department of Medical Biochemistry and Laboratory Diagnostics, General University Hospital in Prague, 12808 Prague, Czech Republic

**Keywords:** *Helicobacter pylori*, human papilloma virus, oropharynx, squamous cell carcinoma, chronic inflammation, incidence

## Abstract

Objective: Oropharyngeal/laryngeal carcinoma are common cancers of the upper aerodigestive system. Human papillomavirus (HPV) is described as the most frequent in the cancer of unknown primary. The presence of *Helicobacter pylori* (HP) in the oral cavity is discussed in some papers. The aim of study: To analyze the incidence of HPV and HP in oropharyngeal/laryngeal cancer persons versus persons with chronic tonsillar inflammation and healthy persons. Methods: The samples were taken in three groups: (1) tissue of oropharynx/larynx cancer (103 specimens); (2) tissue of palatine tonsils (85 specimens); and (3) healthy control group (50 specimens). We analyzed the presence of HP (PCR) and HPV genomic DNA (Sacace HPV High-Risk Screen Real-TM Quant) in the samples. Results: HP was detected in 86 samples (83.5%) and high-risk HPV in 62 samples (60.2%). We found a very high incidence of HP. In the cancer group, HP was detected in 82.5% cases and HPV positivity in 57.8%. In total, 7.2% of the cancer patients were negative for HP and HPV together. In turn, 53.6% of the cancer patients were positive for HP and HPV together. Four cases (4.2%) were positive for HPV only. VacA positivity was detected in 82 (79.6%) of the cancer cases and VacA negativity in 21 (20.4%) if the cancer cases. The incidence of HP in chronic inflammation (*n* = 85) was 65 cases (76.5%) and the incidence of HPV was 38 cases (44.7%). VacA positivity was detected in 59 (69.4%) of the chronic inflammation cases and VacA negativity was found in 26 (30.6%) of the chronic inflammation cases. Regarding the control group, we found HP positivity in 5 cases (11.1%) and HPV positivity in 19 cases (42.2%). There was VacA positivity in 6 cases (50.0%) of the control group. Statistically significantly lower prevalence of HP (*p* < 0.001) and HPV (*p* = 0.006) was found in the control group. Conclusions: We suggest that the palatine tonsils are colonized by HP. In our study, HP was present in oropharyngeal cancer in more cases in comparison with HPV infection. The presence of VacA from HP can have an influence on the human epithelial and immune cells’ regulation ways. Our results do not support idea that the CagA-positive HP is a primary carcinogen in oropharyngeal area.

## 1. Introduction

Oropharyngeal squamous cell carcinoma (SCC) and laryngeal SCC are some of the most common cancers of the upper aerodigestive system. Tobacco and alcohol consumption are the most important risk factors for both these cancers. The other risk factor for oropharyngeal cancer is human papilloma virus (HPV) infection, and this fact was the reason for modification of the tumor classification (TNM classification) and the treatment protocol of oropharyngeal cancer in the last decade. Other contributing factors may include viruses, bacteria, diet type, radiation exposure, gastro-esophageal reflux, occupation, and genetical inheritance [1]. These infections are estimated to cause around 15–20% of all human cancers worldwide. HPV has a well-reported relation, as a carcinogen, to base-of-tongue cancer and tonsillar cancer.

Detection of *Helicobacter pylori* (HP) in saliva [2] supported the idea that it colonizes the oral and oropharyngeal area. Burduk published conclusions that *Helicobacter pylori* can be a carcinogen or co-carcinogen of laryngeal SCC [3].

Sivars et al. described that HPV-positive tonsillar and base-of-tongue SCC have a significantly better prognosis than HPV-negative patients [4]. The most common types causing head and neck squamous cell carcinoma (HNSCC) are HPV 16 and 18. HPV 16 alone is described in more 90% of HPV-positive cases. The HNSCC cancer include the oral cavity, the oropharynx, the nasopharynx, the larynx, the hypopharynx, the lips, and nasal and paranasal sinuses.

An HPV infection influences the presence of cell death molecules 8+ (CD8+) tumor-infiltrating lymphocyte counts and expression of HPV 16 was detected and used as a marker [5]. The oral microbiome is the collective genomes of microorganisms that inhabit the oral and upper aerodigestive tract. [6].

*Helicobacter pylori* was declared a type I carcinogen by the International Agency for Research on Cancer (IARC) in 1994 (IARC Working Group Reports). This bacterium was detected in the oropharynx, too [7,8,9,10].

Chen et al. described a malignant transformation-relevant role of HP in laryngeal precancerous lesions [11]. On the other hand, Pandey et al. described no detection of HP in oral SCC from a cohort of Norwegian and Nepalese patients [12]. Genc et al. detected no signs of the colonization by HP in laryngeal tissues in laryngeal SCC patients [1].

HP was detected in pharyngeal and laryngeal pathologies [10,13,14]. Many authors described the presence of the HP in adenoids and tonsillar tissue depending on polymerase chain reaction (PCR) detection. However, some authors did not detect HP in the same area. This last point should thus be assessed by using certain sampling methods for the detection of HP infection, such as serology analyses and a rapid urease test (RUT), a *Campylobacter*-like organism (CLO) test, PCR genomic DNA detection, and reverse transcription polymerase chain reaction (RT-PCR), among others. The RT-PCR is reported to be the more exact method for detection of HP presence in tissue [10,13,14].

The question about the local immunity depression and/or any other influence on the immunity and cell biology is real. HPV is a carcinogen in the oropharyngeal area. This fact was described in many papers [4,15,16,17,18,19,20]. Relevant studies based on the role of HPV and HP infection in patients with oropharyngeal and/or laryngeal squamous cell carcinoma have not yet been published.

The aim of the study:

(1) To analyze of incidence of infection by HPV and HP in oropharyngeal and laryngeal cancer patients. Can we compare the incidence for cancer patients and chronic tonsillar inflammation to healthy persons?
(2) Analysis of the HPV serotypes present in oropharyngeal and laryngeal cancer patients. Genomic analysis of *Helicobacter pylori* presents in oropharyngeal and laryngeal cancer patients. Determining the incidence of cytotoxin-associated protein (CagA), vacuolating cytotoxin (VacA), and the HPV subtypes in the cancer cohort and chronic inflammation group compared to healthy persons.

## 2. Materials and Methods

This study has a similar methodology as our previous manuscripts based on this grant [14,21,22,23].

The authors declare that the prospective study “Genome of *Helicobacter pylori* and serotype of HPV detected in oropharyngeal and laryngeal cancer and chronic inflammation patients” was conducted with the consent of the Ethics Committee of the Motol University Hospital, Czech Republic—project number IGA NT-11523/6-2010.

The authors declare that the prospective study “Genome of *Helicobacter pylori* and serotype of HPV detected in oropharyngeal and laryngeal cancer and chronic inflammation patients” took place with the consent of the patient. Each patient provided informed consent for accordance into the study. All of them are in accordance with the Helsinki protocols. 

Inclusion parameters [14,21,22,23]: 

Patients with oropharyngeal SCC. The patients were included across all nodal statuses (N0–N3) but no presence of distant metastases. 

Patients with chronic tonsillar inflammation and/or patients with tonsillar infection recurrence were observed minimally two times yearly in the last five years or three or more times in the last two years. No antibiotic therapy was given in the six months preceding surgery. 

The tissue samples were taken after removing the primary tumor by a surgical procedure in cancer patients, and in the chronic inflammation group were taken after tonsillectomy from chronic tonsillitis patients. 

The saliva samples (control group only) were taken from the healthy persons group without any chronic inflammation in their patient history. No signs of cancer were present in the patient’s history [14,21,22,23]. 

Exclusion’s parameters used for all groups:

1. Child and adolescent patients were excluded (age under 18).
2. Pregnant women were excluded.
3. The patients with any of the following: human immunodeficiency virus (HIV), Hepatitis B or C viruses.
4. Any chronic *Salmonella* infections, tuberculosis, or zoonosis were excluded.
5. The patients of any other cancer in history were excluded.
6. The patients with any treatment of the immune system, such as HIV (incl. transplantation of any organs, among others), and patients on immunosuppression therapy (incl. steroids) were excluded.
7. The patients suffering from chronic gastric inflammation and/or ulcers. The persons with a history of from reflux (gastro-esophageal reflux) were excluded.
8. Antibiotic treatment administered within six months preceding the surgery or sample collecting.

Clinical selection of samples was done in three groups:

(a) Tissue of cancer by patients undergoing surgical treatment of oropharyngeal SCC.
(b) Tissue of tonsils by patients undergoing surgical treatment of chronic tonsillitis, suffering one infection yearly for more than 5 years, or 2 infections in the last 3–5 years, or 4–5 infections in the last two years.
(c) For the control group, we used the saliva samples from patients coming for a routine checkup appointment.

### 2.1. Collection and Handing of Samples

Samples were taken from patients suffering from oropharyngeal SCC and chronic tonsillitis (TCR). For the control group, saliva samples from patients with non-oropharyngeal diagnoses were collected [14,21,22,23].

The samples were stored in the Department of Otorhinolaryngology and Maxillofacial Surgery, 3rd Faculty of Medicine Charles University and Military University Hospital Prague. All surgical teams were instructed to handle tissue biopsies according to identical protocols [14,21,22,23].

Collection of tissue specimens was done using sterile instruments at the start of surgery prior to application of any drug disinfection substances into the oral cavity [14,21,22,23].

For collection of samples, we used Remel Microtest^R^ M4RT Collection and Transport Medium. These specimens were transported into the laboratory where they were vortexed, aliquoted, and the nucleic acids isolated using a Roche MagNAPure Compact (Tegimenta AG, Switzerland) automated isolator. The following procedure involved the real-time PCR technique for *Helicobacter pylori* detection and genotyping [14,21,22,23].

We developed and optimized the tests and procedures in co-operation with TIB MolBiol Berlin, Germany. Samples and nucleic acid isolates were stored at −80 °C [14,21,22,23]. The isolates and samples were analyzed for the *Helicobacter pylori* flagellar gene by commercial real-time PCR and commercial real-time PCR screening tests for HPV, based on *fla* gene detection [14,21,22,23].

### 2.2. Detection Techniques

#### 2.2.1. Real-Time PCR Amplification and Genotyping of *Helicobacter pylori*

With regard to genotyping, the *vacA* gene was genotyped from the middle and final regions according to the technology described in the literature [14,21,22,23].

The primers used for PCR detection of HP were used according to van Doorn et al. [16]. For detection of fluorescently labelled hybridization, TaqMan Probes were used [14,21,22,23].

#### 2.2.2. *Helicobacter pylori* Confirmation by Commercial Real-Time PCR Assay

The commercial real-time PCR assay BIORON Real-Line *Helicobacter pylori* Fla-Format Assay (Bioron Diagnostics GmbH., Römerberg, Germany) was used. *Helicobacter pylori* real-time PCR assay probes were used: cagA, vacAs1a, vacAs1b, vacAs2, vacAm1, and vacAm2, comprehensively described in published papers [14,21,22,23].

#### 2.2.3. Human Papilloma Virus DNA Detection

Detection was done by Sacace HPV High-Risk Screen Real-TM Quant Assay (A5, A6, A7 and A9: 16, 18, 31, 33, 35, 39, 45, 51, 52, 56, 58, 59). Assays were performed according to technology instruction and according to published technology [14,21,22,23].

*Helicobacter pylori* assays primer sequences was used for cag AF+, cagAR−, VAF1F+, VAXR−, HPMGF, and HPMGR. This has been comprehensively described in the literature [14,21,22,23].

#### 2.2.4. Statistical Analyses

All statistical analyses were performed using IBM SPSS statistics (version 22.0; SPSS, IBM, Armonk, NY, USA). The data were analyzed using descriptive methods, Fisher’s exact test (two-tailed), and Pearson’s chi-square test. *p*-values < 0.05 were considered statistically significant in all statistical analyses.

## 3. Results

This study analyzed 103 samples taken from squamous cell carcinoma in oropharyngeal area, 85 specimens taken from chronic inflammation of palatine tonsils, and 50 specimens of saliva from healthy persons (control group specimens), using techniques for detection of *Helicobacter pylori* DNA sequences (*cagA* gene, *vacA* gene, and *fla* gene). On the other hand, we analyzed the DNA of the high-risk types of HPV groups A9, A7, and A5/6. The incidence of both HP and HPV is summarized in Table 1.

These data show evidence of HP presence in the cancer tissue in oropharyngeal area, especially in the lymphoid one. We can conclude that the significance of a higher rate of HP presence (83.5%) is based on improved laboratory techniques and methods of sampling, and on the other hand based on a higher incidence of this infection in tissue generally. A higher presence as also observed in chronic tonsillar inflammation patients (76.5%). This incidence is significantly higher compared to the control group (HP incidence 12.0% only). Statistical result: statistically significantly lower prevalence of HP (*p* < 0.001) and HPV (*p* = 0.006) in the control group.

The distribution of CagA in each group is described in Table 2. Mostly, the samples showed the CagA− cytotoxin and only 23.3% of the isolates presented the CagA+ cytotoxin. This protein is considered the main important factor of pathogenicity of HP in the stomach. These data support the low incidence of the CagA+ genotypes in patients with chronic tonsillar inflammation (15.2%), which also only rarely was present in the control group (4.0% only). In contrary, the CagA− genotypes are presented in the cancer and chronic inflammation groups at a similar incidence.

The incidence in Group A was 60.2% and in Group B 61.2%. These data suggest that any other cytotoxin can play a role in the pathogenicity of HP in the oropharyngeal area (see Table 3).

**Figure 1:**
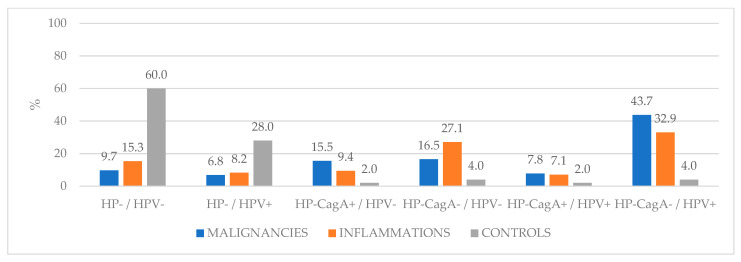
Samples with different co-infection combinations.

The co-infection combinations of HP and/or HPV are described in Table 4. These incidences describe the distribution of both agents in all three groups (Figure 1).

The presence of genotypes of HP in the malignant tumour group (Group A) are displayed in Table 5. The presence of the VacA S1bM1 genotype is the most common, seen in 33.7% of cases in Group A, independent of the CagA genotype. 

On the other hand, VacA S1a,bM1 presents in 41.9% of CagA-negative cases compared to VacAS1,bM2 that presents in 24.4%. This result suggests that the presence of CagA-negative VacA genotypes was higher than the CagA-positive and VacA genotypes generally. This may reinforce the hypothesis that VacA positivity of HP can be the reason for the pathogenicity of HP in oropharyngeal malignancies.

The confirmation of VacA pathogenicity detected by analysis of *Helicobacter pylori* genotypes in the chronic inflammation cases (Group B) is presented in Table 5.

Incidence of the VacA S1bM1 genotype is the most often and presents in 27.7% of cases in Group B, independently of the CagA genotype. On the other hand, in CagA-negative cases, the presentation of VacAS1a,bM1 is in 30.8% of cases compared toVacAS1bM2 in 33.9%. These results suggest that the CagA-negative VacA genotype cases were present in the same incidence (see Table 5, Table 6 and Table 7). This fact can support the hypothesis that the *VacA* gene of *Helicobacter pylori* can play a role in the pathogenicity of *Helicobacter pylori* in oropharyngeal malignancies.

The presence of different genotypes of HP in Group A and Group B compared to the control group suggest the association of HP infection with moderate immunological status of the lymphatic tissue and/or the local immunity (see Table 4). The cancerogenic effect on the tissue in the oropharyngeal area has to be supported by more evidence in the future.

DNA of *Helicobacter pylori* was detected in 86 samples (83.5%), while high-risk HPV in 62 samples (60.2%). The HPV positivity A5/6 and any HP positivity together were detected in 38.8% of cases in Group A. HPV positivity A7 was detected in 16 cases (15.5%) and HPV positivity A9 in 12 cases (11.6%). *Helicobacter pylori* was genotyped as a sole agent in 34 samples (32.98%). HPV A5/6, a7, and A9 was detected as a sole agent in 7 cases (6.8%) only. A high number of papers reported HPV type 16 as the main cancer inductor. HPV type 16 is included the A9 group. In the used tests, we used a specific fluorescent signal for detecting the A9 group. We detected HPV type 19 in the A9 group only in 13 cases out of 60 HPV-positive cases. The A5/A group was detected in 46 cases (44.6%) out of 60 HPV-positive cases. The A7 group was detected in 21 cases (20.4%) out of 60 HPV positive cases.

## 4. Discussion

Chen et al. described the microbiota role in four cancer types (colorectal cancer, head and neck cancer, pancreatic cancer, and lung cancer) [24]. This Baltimore group focused on the evidence of association and causality of microbiota in diseases, specifically, cancers. Therefore, our research shares a component regarding head and neck cancers.

Lopez et al. detected the circulating markers after resection of HNSCC [25]. The analysis included the oncogenic pathogens HPV, Epstein–Barr virus, and HP in 21 patients, with the presence of tumour markers in 52% of patients only (commonly p53 and HPV). This reinforces the well-accepted role of HPV in SCC of the head and neck. De Martel et al. estimated that 16% of new cancers diagnosed in 2008 were attributable to infections [26]. This could indicate that other less studied infections may share a role in the development of head and neck cancers. Ank et al. published a study on oropharyngeal SCC treated by primary RCT, and was able to separate patients in three group (low, intermediate, and high risk) for survival based on HPV status, smoking, tumor staging, and tumor grading [27]. The incidence of HPV and serotypes of HPV described in this study are like the results that were published. The incidence in cancer patient was nearly 60%, but in chronic inflammation (chronic tonsillitis) cases it was much lower. We attribute this finding as evidence of HPV’s influence in carcinogenesis.

The incidence of HP in the tonsil tissue in the chronic inflammation group is of a lower rate than cancer patients.

Payäo et Rasmussen described extra gastric reservoirs of HP in saliva and dental plaque, the tongue, and oropharynx [28]. The authors’ conclusions provide sufficient evidence to support the role of adenoids and tonsils as a reservoir of *Helicobacter pylori*.

The presence of HP in the oropharyngeal area, chronic tonsillitis, and oropharyngeal tumors has been described by us in many publications [8,10,21,22]. This study analyzed 85 samples of chronic tonsillitis (recurrent or chronic tonsillitis). The results showed that 19 cases (22.4%) the samples were *Helicobacter pylori*-negative samples. HP was detected in 77.6% of patients in this group. HPV was positive in 47.1% samples. On the other hand, Hwang et al. published a meta-analysis with no finding of HP colonization in tonsillar tissue [29]. The meta-analysis was done using publications that were published from 1997 to 2014 [29]. Siupsinskiene et al. analyzed 62 patients with chronic tonsillitis and 35 with hypertrophy of tonsillar tissue [30]. The conclusions were that the HP infection and chronic tonsillitis and/or laryngopharyngeal reflux present coincidence and the infection can be a reason for this. The presence of HP in the adenoid tissue and its association to reflux episodes was described [23].

The role of HP infection in adenoid and tonsillar tissue is unclear yet. The incidence of HP in tonsils is not strong evidence of the influence of HP to immune cells in the oropharynx. On the other hand, the presence of CagA-positive HP in stomach area associates with a cancerogenic effect. This fact can relate to some influence on the local immunity in the tonsils and/or oropharyngeal area. The sharp difference in HP presence in the oropharyngeal area published in many papers depends on methods of HP detection. The data in our study support that the “gold standard” for oropharyngeal area is the PCR detection of the HP. Naserpour Farivar et al. described the colonization of tonsils in chronic tonsillitis patients by HP [31]. These authors formulated conclusions that the existence of *Helicobacter pylori* in tonsillar tissue samples of patients with chronic tonsillitis is controversial.

The results of this study showed a very high incidence of HP detected by PCR resp. rt-PCR. In the cancer group, the HP was detected in 82.5% cases. In the same group there was HPV positivity in 57.8%. Only 7.2% of cancer patients were negative both for *Helicobacter pylori* and HPV. To the contrary, 53.6% of cancer patients were positive both for HP and HPV. Only four cases (4.2%) were positive for HPV only.

If we compare these results to the control group of saliva analyses of healthy persons, we can see that HP positivity was detected in 5 cases (11.1%) and HPV positivity in 19 cases (42.2%). A saliva sample was used for the control group because no tissue samples could be taken in healthy patients. The HP detection in saliva was described in the literature [32]. The use of a saliva sample is supported by studies that demonstrate the possibility of using HPV16 DNA detection in saliva as a potential screening tool for the detection of HPV-positive oropharyngeal SCC [33]. On the other hand, it can be assumed that the accuracy of HPV or HB detection in saliva may be lower than from tissue samples. We take this fact into account in our study.

We suggest that the fact about the incidence of HP in the oropharyngeal area is described in the present study.

We have analyzed the HP genome, respectively, the *vacA* and *cagA* genes. Our analysis focused on the incidence of the CagA and VacA cytotoxins. The presence of CagA-positive HP was in 23 cases (23.7%). CagA was described as the main carcinogenic factor in the stomach. Hatakeyama described the main carcinogenic process in stomach epithelial cells by bacteria through the effect of tyrosine phosphorylation at the Glu-Pro-Ile-Tyr-Ala (EPIYA) [34]. This way, the *cagA* gene-encoded CagA protein is delivered into the gastric epithelial cells. The CagA intracellular effect promotes the neoplastic transformation in epithelial cells in the stomach. The role VacA in the epithelial human cells has not been described yet. In the literature described, some have studied an association between the *vacA* s1/m1 strains and gastric adenocarcinoma [35,36]. The role of VacA in immunity was described completely by Farnaz Fahmi et al. [37]. The VacA activity is in the interaction with the vacuole surface and developing of vacuoles in the cells. On the other hand, this cytotoxin has an influence on the secretion of CytC for starting the apoptotic activity in the cells. VacA was classified as a pore-forming cytotoxin, and many of its effects on epithelial cells are influenced by the channel functions in the intracellular space. HP VacA cytotoxin facilitates bacteria against the defense system. The toxin has multiple effects in epithelial cells and immune cells such as T lymphocytes, B lymphocytes, macrophages, and dendritic cells [38]. The activity of this pore-forming cytotoxin in the immune system can be modified by the cytokine activity in the cells and/or can influence apoptotic activity, too [39].

The significant difference among the presence of HP in the respective group and the higher incidence of *Helicobacter pylori* than HPV lead to more questions. We cannot asign HP to be a carcinogen for the oropharyngeal area yet. The evidence requires further studies. The cancerogenic effect of HPV has been declared in the literature, with strong evidence.

## 5. Conclusions

Considering our data, we suggest the revision of the theory on HPV-induced oropharyngeal cancer. Our results should support the hypothesis about the influence of HP (VacA) on local immunity. It seems evident that HP is a long-term resident in the oropharyngeal area and tonsils. We suggest that HP should be seen as a contributor who could switch on the process, resulting in cancerogenesis.

We believe that the results in this study support the following conclusions:

We suggest that the tonsils (oropharyngeal area) can be colonized by *Helicobacter pylori*. The high frequency of detected DNA of HP in the tissue of cancers and tonsillar tissue is the reason for these conclusions that HP is a resident in the oropharyngeal area. 

The presence of HP in oropharyngeal cancer is real in more cases compared to HPV infection.

- The majority of HPV-infected patients has an HP infection at the same time in the same sample.
- The presence of HPV in oropharyngeal cancer patients alone is rare.
- Data in the study suggest that HP can influence local immune cells and may cause a decrease in immunity towards HPV and/or any other infectious agents.
- The CagA-positive HP was detected in less cases. This result of the study does not support the idea that CagA-positive HP is a primary carcinogen in the oropharyngeal area.

The high number of HP patients in the oropharyngeal cancer group compared to the HPV infection must be explained in the next study.

The incidence of HP infection and the presence of different genotypes suggest the association of infectious agents with oropharyngeal cancer.

## Figures and Tables

**Table 1:** Absolute and percent occurrence of *Helicobacter pylori* and human papillomaviruses in the malignancy, chronic inflammation, and control groups.

	Total	HP+	%	*p*-Value	HPV+	%	*p*-Value
Malignancy Group A	103	86	83.5	<0.001	62	60.2	0.006
Inflammation Group B	85	65	76.5		38	44.7	
Control Group C	50	6	12.0		17	34.0	

HP—*Helicobacter pylori*; HPV—human papilloma virus; +—positive detection.

**Table 2:** Distribution of the CagA+ and CagA− genotypes of *Helicobacter pylori* in the respective groups.

	Total	HP+	CagA+	% of Total	CagA−	% of Total
Malignancy Group A	103	86	24	23.3	62	60.2
Inflammation Group B	85	65	13	15.3	52	61.2
Control Group C	50	6	2	4.0	4	8.0

HP—*Helicobacter pylori*; CagA—cytotoxin-associated protein; +—positive detection; −—negative detection.

**Table 3:** Percentage of the CagA+ genotype in samples with *Helicobacter pylori* detected.

	Total HP+	CagA+	%	CagA−	%	*p*-Value
Malignancy	86	24	27.9	62	72.1	0.482
Group A						
Inflammation	65	13	20.0	42	80.0	
Group B						
Control	6	2	33.3	4	66.7	
Group C						

HP—*Helicobacter pylori*; CagA—cytotoxin-associated protein; +—positive detection; −—negative detection.

**Table 4:** Samples with different co-infection combinations.

**Malignancy (*n* =103)**	**Total**	**%**
HP−/HPV−	10	9.7
HP−/HPV+	7	6.8
HP-CagA+/HPV-	16	15.5
HP-CagA−/HPV−	17	16.5
HP-CagA+/HPV+	8	7.8
HP-CagA−/HPV+	45	43.7
**Chronic Inflammation (*n* = 85)**	**Total**	**%**
HP−/HPV−	13	15.3
HP−/HPV+	7	8.2
HP-CagA+/HPV−	8	9.4
HP-CagA−/HPV−	23	27.1
HP-CagA+/HPV+	6	7.1
HP-CagA−/HPV+	28	32.9
**Control Group (*n* = 50)**	**Total**	**%**
HP−/HPV−	30	60.0
HP−/HPV+	14	28.0
HP-CagA+/HPV−	1	2.0
HP-CagA−/HPV−	2	4.0
HP-CagA+/HPV+	1	2.0
HP-CagA−/HPV+	2	4.0

HP—*Helicobacter pylori*; HPV—human papilloma virus; CagA—cytotoxin-associated protein; +—positive detection; −—negative detection.

**Table 5:** Distribution of different *Helicobacter pylori* genotypes in positive samples of the respective groups (malignancy, *n* = 86).

CagA Protein Gene Detection	Genotype of VacA Cytotoxin	DETECTED (N)	%
POSITIVE	VacA S1a M1	4	4.7
POSITIVE	VacA S1a M2	5	5.8
POSITIVE	VacA S1b M1	10	11.6
POSITIVE	VacA S1b M2	4	4.7
POSITIVE	VacA S2 M1	1	1.2
POSITIVE	VacA S2 M2	0	0.0
NEGATIVE	VacA S1a M1	17	19.8
NEGATIVE	VacA S1a M2	11	12.8
NEGATIVE	VacA S1b M1	19	22.1
NEGATIVE	VacA S1b M2	10	11.6
NEGATIVE	VacA S2 M1	5	5.8
NEGATIVE	Vac A S2 M2	0	0.0

**Table 6:** Distribution of the different *Helicobacter pylori* genotypes in positive samples of the chronic tonsillitis groups: (a) inflammation, *n* = 65.

CagA Protein Gene Detection	Genotype of VacA Cytotoxin	DETECTED (N)	%
POSITIVE	VacA S1a M1	8	12.3
POSITIVE	VacA S1a M2	1	1.5
POSITIVE	VacA S1b M1	7	10.8
POSITIVE	VacA S1b M2	1	1.5
POSITIVE	VacA S2 M1	0	0.0
POSITIVE	VacA S2 M2	0	0.0
NEGATIVE	VacA S1a M1	9	13.9
NEGATIVE	VacA S1a M2	10	15.4
NEGATIVE	VacA S1b M1	11	16.9
NEGATIVE	VacA S1b M2	12	18.5
NEGATIVE	VacA S2 M1	6	9.2
NEGATIVE	VacA S2 M2	0	0.0

**Table 7:** Distribution of the different *Helicobacter pylori* genotypes in positive samples of the control group: (b) controls, *n* = 12.

CagA Protein Gene Detection	Genotype of VacA Cytotoxin	DETECTED (N)
POSITIVE	VacA S1a M1	1
POSITIVE	VacA S1a M2	
POSITIVE	VacA S1b M1	
POSITIVE	VacA S1b M2	
POSITIVE	VacA S2 M1	1
POSITIVE	VacA S2 M2	
NEGATIVE	VacA S1a M1	1
NEGATIVE	VacA S1a M2	1
NEGATIVE	VacA S1b M1	1
NEGATIVE	VacA S1b M2	1
NEGATIVE	VacA S2 M1	
NEGATIVE	VacA S2 M2	
